# Melatonin suppresses abnormal hyperexcitability of hippocampal CA1 neurons in early-stage oxygen-glucose deprivation

**DOI:** 10.3389/fphar.2026.1836828

**Published:** 2026-05-26

**Authors:** Yang Cui, Qi Wang, Chao Liu, Heng Zhou

**Affiliations:** Jiangsu Key Laboratory of Brain Disease and Bioinformation, Xuzhou Medical University, Xuzhou, China

**Keywords:** hippocampal CA1 neurons, melatonin, neuronal excitability, oxygen-glucose deprivation, patch-clamp recording

## Abstract

Excitotoxicity in the early stages of ischemia is a primary trigger of neuronal injury. Literature has indicated the potential neuroprotective effects of melatonin against ischemic hypoxic injury, but the direct electrophysiological mechanisms underlying its regulation of neuronal excitability remain not fully understood. In this study, using an *in vitro* oxygen-glucose deprivation (ODG) model to mimic ischemic-hypoxic injury, we applied whole-cell patch-clamp recording in mouse brain slices to investigate the effects of melatonin on the excitability of hippocampal CA1 neurons at the early stage of OGD. After 10 min of OGD, CA1 neurons developed marked membrane depolarization, burst firing, and dramatically increased the frequency of spontaneous excitatory postsynaptic current (sEPSC) while reducing the frequency of spontaneous inhibitory postsynaptic current (sIPSC). Compared with the control group, the OGD operation significantly elevated the firing frequency of action potential, prolonged the action potential half-width, and shortened the latency of evoked action potentials. Melatonin treatment significantly attenuated these abnormal changes: it reduced firing frequency, reversed action potential half-width, and prolonged latency of evoked action potentials. Additionally, melatonin alleviated the OGD-induced abnormality in the frequency of sEPSC and sIPSC, without affecting their amplitude. Thus, melatonin suppresses the OGD-induced abnormal hyperexcitability of neurons and improves action potential kinetics during early ischemic-hypoxic injury.

## 1 Introduction

Ischemic stroke ranks as one of the leading causes of death and long-term disability globally, imposing a heavy medical and economic burden on individuals and healthcare systems (Feigin et al., 2025). The core pathological event of ischemic stroke is the reduction or complete interruption of cerebral blood flow perfusion, which triggers a cascade of pathological reactions leading to extensive neuronal death and permanent neurological dysfunction (Yu et al., 2021; Feigin et al., 2025). Given the narrow therapeutic time window of current reperfusion therapies, including intravenous thrombolysis and endovascular thrombectomy, there is an urgent need to develop effective neuroprotective agents that can intervene in early neuronal injury to improve clinical outcomes (Tsivgoulis et al., 2023; Zhang et al., 2024b), as well as the mechanisms of early ischemic-hypoxic neuronal injury under some conditions (Yu et al., 2015; Xu et al., 2025; Yang et al., 2025).

Multiple mechanisms contribute to ischemic-hypoxic brain injury, including oxidative stress, neuroinflammation, metabolic acidosis, and excitotoxicity (Zhang et al., 2024b). Among these, excitotoxicity is considered the core early event that triggers irreversible neuronal damage within a few minutes after blood flow interruption (Neves et al., 2023). After oxygen and glucose deprivation, there is a sharp decline in neurons’ ability to produce adenosine triphosphate (ATP), subsequently causing failure of the cell membrane sodium-potassium pump (Na+/K + -ATPase) function, resulting in sustained neuronal membrane depolarization (Schoknecht et al., 2025). This disturbance process rapidly leads to sustained neuronal membrane depolarization, triggering the opening of voltage-gated ion channels, and promoting the massive release of excitatory transmitters like glutamate, and extensively activates their postsynaptic receptors, eventually leading to intracellular calcium overload and subsequent neuronal injury (Wang et al., 2025). Therefore, inhibiting abnormal neuronal hyperexcitability in the early stage of ischemia is a key therapeutic strategy to rescue these sensitive neurons (Fujioka et al., 2004; Xu et al., 2025).

Melatonin (N-acetyl-5-methoxytryptamine), an endogenous indoleamine hormone primarily synthesized and secreted by the pineal gland (Chlubek and Sikora, 2020), has been identified as a promising neuroprotective agent for ischemic brain injury (Patiño et al., 2016; Zang et al., 2020). It can easily cross the blood-brain barrier and is well-tolerated in clinical applications, making it an ideal candidate drug for treating related central nervous system diseases (Alghamdi, 2018; Mineiro et al., 2024). Previous studies have revealed some protective mechanisms of melatonin, including anti-oxidative stress, anti-inflammation, and anti-apoptosis (Zang et al., 2020; Kolodziejska et al., 2025). Recently, with the deepening of research on the pathological mechanisms of ischemic stroke, melatonin, as a multi-effective endogenous neuroprotectant, has attracted increasing attention for its role in combating ischemic-hypoxic injury (Andrabi et al., 2015; Ramos et al., 2017; Zhang et al., 2024a). It can modulate ion channel activity and enhance GABAergic inhibitory transmission, as well as some signaling pathways, to antagonize glutamate excitotoxicity (Ayar et al., 2001; Cheng et al., 2012; Choi et al., 2014; Pissas et al., 2024), thereby inhibiting neuronal over-depolarization.

However, the direct electrophysiological evidence about how melatonin affects neuronal excitability during the very early stage of OGD remains lacking. In this study, we used whole-cell patch-clamp recording to systematically investigate the regulatory effects of melatonin on neuronal excitability and synaptic transmission in hippocampal CA1 neurons at the early stage of OGD, aiming to provide a solid experimental basis for the clinical application of melatonin in early ischemic stroke intervention.

## 2 Materials and methods

### 2.1 Animals and instruments

C57BL/6 mice, 8–12 weeks old, weighing (22 ± 4) g, were purchased from Jiangsu GemPharmatech Co., Ltd. (Production License No.: SCXK (Su) 2025-0013; Laboratory Animal Use License No.: SYXK (Su) 2025-0059). All animals were group housed in ventilated cages with free access to water and food, a 12/12-h light/dark cycle, and a thermoregulated environment. All procedures followed the institutional guidelines and were approved by the Animal Ethics Committee of Xuzhou Medical University (Approval No.: 202209S078).

MultiClamp 700B amplifier, Digidata 1550B data acquisition system, pClamp 10.7 software (Molecular Devices/Axon, United States of America); P-97 micropipette puller, MP-225 micromanipulator (Sutter Instrument, United States of America); Leica VT1200S vibratome (Leica, Germany); IX73 inverted fluorescence microscope (Olympus, Japan); PC-10 glass capillary tubes (Sutter Instrument, United States of America); G-1 glass microelectrodes (Warner Instruments, United States of America); Ultrapure water system (Millipore, United States of America).

### 2.2 Regents

Melatonin (purity ≥98%, MedChemExpress, United States of America); Sodium pentobarbital (Sigma-Aldrich, United States of America); Sodium chloride, Potassium chloride, Sodium dihydrogen phosphate, Sodium bicarbonate, Glucose, Magnesium chloride, Calcium chloride (all analytical grade, Shanghai Sigma-Aldrich, China); D-Anhydrous glucose (Sigma-Aldrich, United States of America); Sucrose (Sigma-Aldrich, United States of America). Artificial cerebrospinal fluid (aCSF) composition: NaCl 126, KCl 2.5, NaH_2_PO_4_ 1.25, MgCl_2_ 2, CaCl_2_ 2, NaHCO3 26, Glucose 10 (unit: mmol·L^-1^), pH 7.3∼7.4, saturated with 95% O_2_ + 5% CO_2_ gas mixture. Action potential and sEPSC were recorded using pipettes filled with an internal solution containing (in mmol/L): 10 HEPES, 4 NaCl, 140 K-methylsulfate, 4 MgATP, 0.2 EGTA, 0.3 Na_3_GTP, and 10 phosphocreatine at a holding potential of −70 mV. sIPSC was recorded with a CsCl-based intracellular solution containing (mM): 135 CsCl, 10 HEPES, 0.2 EGTA, 2 Na_2_ATP, 0.3 Na_3_GTP, and 10 glucose. pH adjusted to 7.2∼7.3 with KOH, and the osmolality was measured using a freezing-point osmometer and maintained between 290–300 mOsm. A stock solution of melatonin was prepared in DMSO and diluted in ACSF on the day of the experiment to a final concentration of 20 μM. The maximal DMSO concentration in the ACSF was maintained below 0.2% (v/v).

### 2.3 Preparation of hippocampal slices

Mice were anesthetized via intraperitoneal injection of sodium pentobarbital (60 mg/kg) and rapidly decapitated. The whole brain was quickly removed and immersed in ice-cold oxygenated aCSF. Hippocampal coronal slices (250 μm thick) were cut using a vibratome, then incubated in oxygenated aCSF at 28 °C for 1 h for recovery. After incubation, slices were transferred to a recording chamber continuously perfused with oxygenated normal aCSF (2∼3 mL/min) at room temperature. For electrophysiological recordings, pyramidal neurons in the hippocampal CA1 region were visually identified based on their location and characteristic morphology. Glass micropipettes (3∼5 MΩ resistance when filled with intracellular solution) were positioned onto the neuron surface. A gigaseal (>1 GΩ) was formed by gentle suction, then the membrane was ruptured to establish whole-cell configuration. After 3∼5 min of stabilization, action potentials were recorded in current-clamp mode. In voltage-clamp mode, sEPSCs and sIPSCs were recorded at holding potentials of −70 mV and 0 mV, respectively. All patch-clamp procedures were conducted at room temperature (22 °C∼25 °C) following our previously published protocol (Liu et al., 2017).

### 2.4 OGD model, grouping and drug intervention

Mice were randomly divided into four groups: (1) Control group: continuously perfused with normal oxygenated aCSF; (2) Melatonin alone group: perfused with normal aCSF containing 20 μM melatonin; (3) OGD group: After baseline recording, the perfusion solution was rapidly switched to glucose-free OGD-aCSF (where glucose was replaced with an equimolar concentration of sucrose to maintain osmolality). This OGD-aCSF was continuously bubbled with 95% N_2_ and 5% CO_2_ to achieve hypoxia. Neurons were perfused with OGD-aCSF for 10 min, and electrophysiological responses were recorded; (4) OGD + Melatonin group: After a baseline recording in normal oxygenated ACSF, the perfusion was simultaneously switched to OGD-ACSF containing 20 μM melatonin. After 10 min of co-perfusion with this solution, the electrophysiological responses were recorded. This protocol ensured that melatonin treatment was synchronous with the onset of oxygen-glucose deprivation.

### 2.5 Statistical analysis

Data were processed and plotted using GraphPad Prism 9.1.0. Results are expressed as mean ± standard error of the mean. Statistical analysis between groups was performed using Two-way ANOVA followed by *post hoc* comparison, accordingly. ^*^ *P* < 0.05 was considered statistically significant.

## 3 Results

### 3.1 Melatonin partially stabilizes membrane potential and inhibits OGD-induced spontaneous hyperexcitability in hippocampal CA1 neurons

**FIGURE 1:**
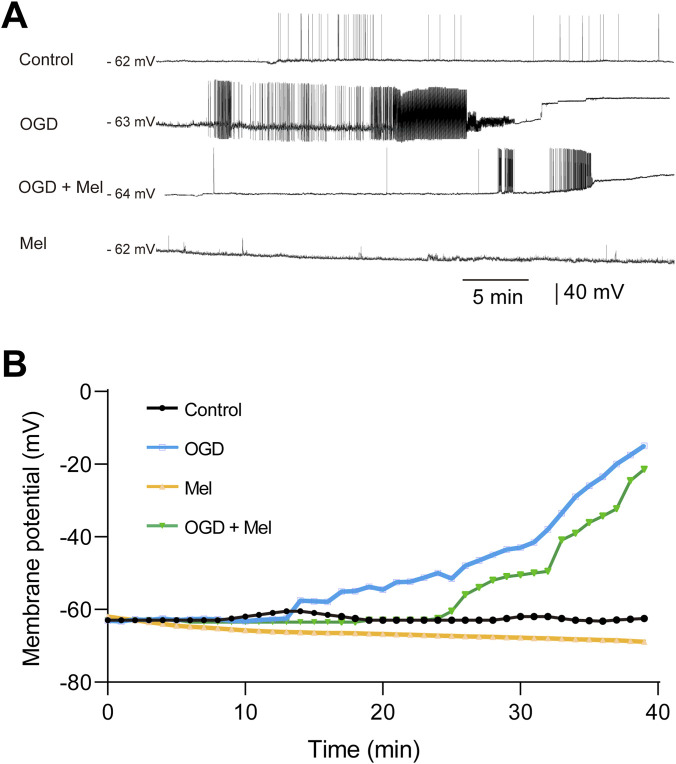
Effect of melatonin on membrane potential changes of hippocampal CA1 neurons. **(A)** Representative recording traces of the membrane potential of hippocampal CA1 pyramidal neurons in each group. **(B)** The curves of neuronal membrane potential changes over time across groups (Repeated 4 times for each).

Using whole-cell patch-clamp in current-clamp mode, continuous recordings of the membrane potential of neurons in hippocampal CA1 were made. In the control group (perfused with normal aCSF), neurons maintained a stable resting membrane potential, with only rare low-frequency spontaneous action potentials. In the melatonin alone group, neurons showed slight membrane hyperpolarization, indicating that melatonin itself has an inhibitory effect on basal neuronal excitability under normal physiological conditions. Differently, neurons elicited rapid and marked membrane depolarization, accompanied by a significant increase in spontaneous action potential frequency and dense burst firing, a typical manifestation of early excitotoxicity, when these brain slices were exposed to the OGD environment for 10 min (OGD). However, melatonin pretreatment significantly delayed OGD-induced membrane depolarization, reduced spontaneous firing frequency, and eliminated dense burst firing (OGD + melatonin) (Figure 1). These results confirmed that melatonin partially stabilized membrane potential and inhibited OGD-induced abnormal spontaneous hyperexcitability.

### 3.2 Melatonin attenuated OGD-induced evoked action potential abnormalities. Increased firing frequency and prolonged half-width

**FIGURE 2:**
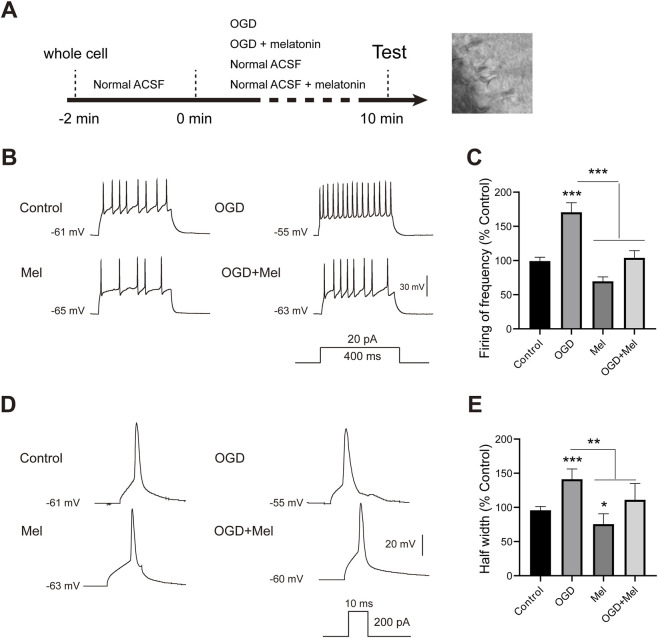
Effect of melatonin on evoked action potentials of hippocampal CA1 neurons. **(A)** Experimental timeline of the OGD and drug application protocol (left), and a representative image of a patched hippocampal CA1 pyramidal neuron (right). **(B)** Representative recording traces of evoked action potentials of hippocampal CA1 pyramidal neurons in each group (evoked by a 20 pA, 400 ms depolarizing current injection). **(C)** Statistical graph of action potential firing frequency in each group (Two-way ANOVA: F _(3, 27)_ = 23.16, *P* < 0.001, *post hoc* comparison, OGD vs. Control, *P* < 0.001; Mel vs. Control, *P* > 0.05; OGD + Mel vs. OGD, *P* < 0.001; OGD vs. Mel, *P* < 0.001; OGD + Mel vs*.* Control, *P* > 0.05; n = 10 neurons/each bar). **(D)** Representative recording traces of single evoked action potentials of hippocampal CA1 pyramidal neurons in each group. **(E)** Statistical graph of action potential half-width across groups (Two-way ANOVA: F _(3, 27)_ = 31.43, *P* < 0.001, *post hoc* comparison, OGD vs. Control, *P* < 0.001; Mel vs. Control, *P* < 0.05; OGD + Mel vs. OGD, *P* < 0.01; OGD + Mel vs. Control, *P* > 0.05; n = 10 neurons/each bar). OGD: oxygen-glucose deprivation; Mel: melatonin. Bar graphs are represented as mean ± S.E.M. *P < 0.05, **P < 0.01 and ***P < 0.001.

To assess the effects of melatonin on neuronal excitability under the stimulus condition, the evoked action potentials by a 20 pA, 400 ms current pulse injection were recorded., and data were analyzed as the percentage change from the first (baseline) stimulus to the second stimulus applied 10 min after the OGD (Figures 2A,B). The Group data indicated that melatonin alone showed an inhibitory effect, reducing the firing frequency to near control levels, while OGD treatment significantly increased the firing frequency of evoked action potentials. Meanwhile, melatonin effectively antagonized the OGD-induced neuronal hyperexcitability, restoring the firing frequency essentially to normal levels, showing a significant difference compared to the OGD group, when OGD was combined with melatonin treatment (Figure 2C). So, melatonin can effectively reverse the OGD-induced high-frequency firing of evoked action potentials.

Furthermore, the half-width of an action potential is an important parameter for describing its shape, and it refers to the duration when the action potential decays to half of its peak amplitude, indicating the properties of action potential kinetics. For this purpose, we analyzed the group effects on the individual action potential waveform (Figure 2D). Compared to the control group, OGD treatment significantly prolonged the action potential half-width, suggesting that the ischemic-hypoxic state slows neuronal action potential repolarization. The action potential half-width in the melatonin-alone group was significantly shorter than in the control group, indicating that melatonin shortens action potential duration, promotes membrane repolarization, and thereby reduces neuronal excitability. In the OGD + Melatonin group, the action potential half-width was significantly reduced compared to the OGD group, suggesting that melatonin can partially reverse the OGD-induced prolongation of action potentials, normalizing action potential kinetics (Figure 2E). Together, these results indicated that melatonin can ameliorate the abnormal action potential kinetics caused by OGD.

### 3.3 Melatonin ameliorates OGD-induced shortening of evoked action potential latency

**FIGURE 3:**
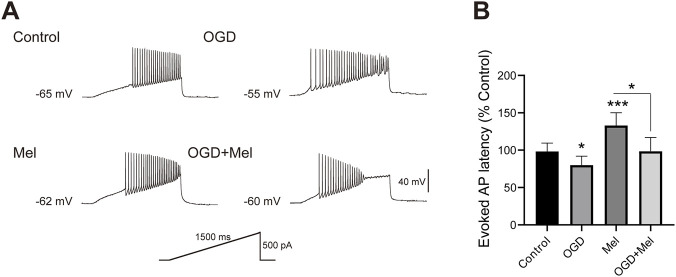
Effect of melatonin on the action potential latency of hippocampal CA1 neurons. **(A)** Representative recording traces of evoked action potentials under ramp current stimulation in each group. **(B)** Statistical graph of evoked action potential latency in each group (Two-way ANOVA: F _(3, 27)_ = 32.86, *P* < 0.001, *post hoc* comparison, OGD vs. Control, *P* < 0.05; Mel vs. Control, *P* < 0.001; Mel vs. OGD + Mel, *P* < 0.05; OGD + Mel vs. Control, *P* > 0.05; n = 10 neurons/each bar). Bar graphs are represented as mean ± S.E.M. *P < 0.05 and ***P < 0.001.

Next, the latency of evoked action potentials under depolarizing current stimulation was recorded (Figure 3A). Compared to the control group, OGD treatment significantly shortened the latency of evoked action potentials. The melatonin-alone group showed a significantly prolonged latency compared to the control. In the OGD + Mel group, the action potential latency was significantly increased compared to the OGD group (Figure 3B). The results indicated that melatonin can partially ameliorate the OGD-induced shortening of evoked action potential latency.

### 3.4 Melatonin differentially modulates spontaneous postsynaptic currents under OGD

**FIGURE 4:**
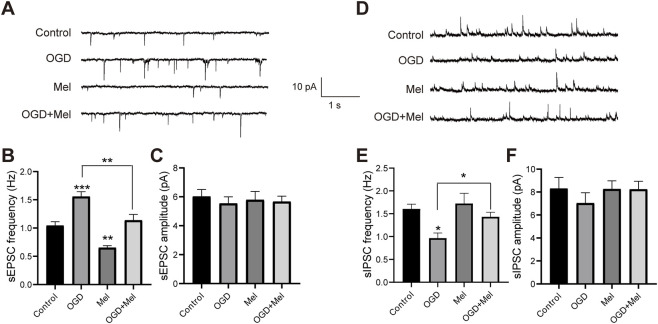
Effect of melatonin on spontaneous postsynaptic currents of hippocampal CA1 neurons. **(A)** Representative recording traces of sEPSC of hippocampal CA1 pyramidal neurons in each group. **(B)** Statistical graphs of sEPSC frequency (Two-way ANOVA: F _(3, 21)_ = 25.59, *P* < 0.001, *post hoc* comparison, OGD vs. Control, *P* < 0.001; Mel vs. Control, *P* < 0.01; OGD vs. OGD + Mel, *P* < 0.01; OGD + Mel vs. Control, *P* > 0.05; n = 6 neurons/each bar), and **(C)** sEPSC amplitude (Two-way ANOVA: F _(3, 21)_ = 0.14, *P* = 0.931). **(D)** Representative recording traces of sIPSC of hippocampal CA1 neurons in each group. **(E)** Statistical graphs of sIPSC frequency (Two-way ANOVA: F _(3, 21)_ = 4.691, *P* < 0.05, *post hoc* comparison, OGD vs. Control, *P* < 0.05; Mel vs. Control, *P* < 0.05; OGD vs. OGD + Mel, *P* < 0.05; OGD + Mel vs. Control, *P* > 0.05; n = 6 neurons/each bar), and **(F)** amplitude (Two-way ANOVA: F _(3, 21)_ = 0.15, *P* = 0.923). Bar graphs are represented as mean ± S.E.M. *P < 0.05, **P < 0.01 and ***P < 0.001.

Finally, we evaluated the effects of melatonin on spontaneous postsynaptic currents under OGD conditions, including spontaneous excitatory postsynaptic currents (sEPSCs) and spontaneous inhibitory postsynaptic currents (sIPSCs). Compared to the control groups, the OGD significantly increased the frequency of sEPSCs in hippocampal CA1 neurons. The group with melatonin treatment, including melatonin alone and OGD + melatonin, showed significantly reduced sEPSCs frequency compared to the OGD groups. Differently, the amplitude of sEPSCs was comparable across all designed groups (Figures 4A–C). Correspondingly, the frequency of sIPSCs was significantly reduced under the OGD condition, which was rescued with melatonin treatment and approximated the control level. Similarly, neither intervention affected sIPSC amplitude across all groups (Figures 4D–F). These results indicated melatonin counteracts excitotoxicity by enhancing inhibitory synaptic transmission and reducing excitatory synaptic transmission, corroborating previous findings on melatonin’s regulation of neurotransmitters (Cheng et al., 2012).

## 4 Discussion

In this study, we employed whole-cell patch-clamp recording to assess the protective effects of melatonin against ischemic-hypoxic injury in hippocampal CA1 neurons during the early stage of the OGD (10 min). In the spontaneous state, OGD induced marked membrane depolarization and burst firing along with an increase in sEPSCs frequency and a reduction in sIPSC frequency. In the evoked state, OGD dramatically elevated action potential firing frequency, prolonged action potential half-width, and shortened evoked action potential latency. Importantly, melatonin treatment significantly attenuated all these abnormal changes. It reduced firing frequency, normalized the prolonged action potential half-width, prolonged the shortened evoked action potential latency, and corrected OGD-induced abnormalities in sEPSC and sIPSC frequency.

Ischemic-hypoxic injury is an important pathological basis for functional impairment induced by ischemic stroke. Following cerebral blood flow interruption, immediate insufficiency of oxygen and glucose supply in brain tissue triggers a series of pathological cascades leading to neuronal damage and death (Zhang et al., 2024b). Among these, excitotoxicity is a crucial mechanism of early neuronal injury in ischemia (Neves et al., 2023; Schoknecht et al., 2025; Wang et al., 2025). Evidence suggests that melatonin has a protective effect against excitatory injury caused by early ischemic stroke (Ramos et al., 2017), but the specific manner in which melatonin affects related neuronal excitability remains unclear. Using an OGD model and patch-clamp electrophysiology, we confirmed the protective effect of melatonin against neuronal excitotoxicity in the hippocampus, a brain region that is highly sensitive to ischemia and hypoxia (Tanaka et al., 1997; Yu et al., 2021). CA1 neurons exhibit significant membrane depolarization and abnormal discharge activity, which is considered an important manifestation of early excitotoxicity under OGD conditions (Tanaka et al., 1997). This study shows that under OGD conditions, mouse hippocampal CA1 neurons exhibit obvious membrane depolarization accompanied by burst firing, indicating a hyperexcitable state. This result is consistent with previous reports on ischemia/hypoxia-induced neuronal hyperexcitability (Neves et al., 2023), thus revalidating the value of this model in related electrophysiological research.

Under OGD conditions, the firing frequency of evoked action potentials in CA1 neurons significantly increased, and melatonin intervention significantly reduced this frequency, suggesting that melatonin could inhibit OGD-induced neuronal hyperexcitability. This result aligns with previous reports that melatonin can reduce abnormal discharges and neuronal hyperexcitability in hippocampal neurons (Tanaka et al., 1997). Furthermore, this study analyzed changes in the action potential waveform. The results showed that OGD significantly prolonged the action potential half-width, and melatonin treatment partially reversed this change. Considering that changes in action potential half-width are often related to alterations in voltage-dependent potassium or calcium channel function, melatonin may improve neuronal action potential kinetics by modulating the activity of related ion channels (Al Dera et al., 2022). This study also found that the latency of evoked action potentials was significantly shortened under OGD conditions, and melatonin treatment partially restored this change. Action potential latency reflects neuronal responsiveness to stimulation, and its shortening typically indicates increased neuronal excitability (Tanaka et al., 1997). The prolongation of this latency by melatonin suggests it may reduce neuronal excitability by modulating neuronal membrane ion channels or synaptic transmission. Additionally, melatonin significantly suppressed the frequency of spontaneous excitatory postsynaptic currents under OGD conditions, also hinting that it may affect neuronal excitability through mechanisms like neurotransmitter regulation. These results further support the view that melatonin exerts neuroprotective effects in the early stages of ischemia-hypoxia by regulating neuronal excitability.

Under normal conditions, melatonin reduces the synaptic efficiency and/or excitability of hippocampal neurons most likely via melatonin MT2 receptor (Hogan et al., 2001), and suppresses hippocampal long-term potentiation, a plasticity dependent on elevated excitability and synaptic transmission (Wang et al., 2005). Furthermore, the inhibitory effects of melatonin have profound physiological relevance, which is likely conserved across multiple brain conditions (Eslamizade et al., 2024; Karimani et al., 2025). For amyloid pathology, amyloid-β directly upregulates transcription of melatonin receptors (MT1 and MT2), and induces hippocampal neuronal hyperexcitability, which is attenuated by melatonin treatment via modulation of after-hyperpolarization and Ih currents (Eslamizade et al., 2024). In aging-associated conditions, melatonin alleviates hippocampal and prefrontal hyperexcitability, improving sleep-related oscillations and enhancing memory (Karimani et al., 2025). In contrast, under seizure conditions, inactivation of hippocampal melatonin receptors could prolong seizure latency and protect animals against seizure development in a GABAA receptor-dependent manner (Stewart and Leung, 2005). Furthermore, the inhibitory effects of melatonin have been proven to rely on the MT2 receptor under certain conditions (Hogan et al., 2001; Wang et al., 2005). Taken together, these findings support that melatonin acts as a consistent endogenous regulator of hippocampal excitability, and the suppression of hyperexcitability in our study extends this fundamental regulation under OGD.

However, this study has certain limitations. First, the experiments primarily observed the effects of melatonin on neuronal excitability from an electrophysiological perspective, without further exploring the specific ion channels or molecular mechanisms involved. Second, this study used an *in vitro* brain slice OGD model. Further validation of melatonin’s neuroprotective effects is needed using *in vivo* ischemia models and *in vivo* electrophysiological techniques (Zhou et al., 2019), combined with ion channel blockers or molecular biology techniques to systematically elucidate the neural mechanisms by which melatonin regulates neuronal excitability.

In conclusion, this study, using whole-cell patch-clamp electrophysiology, confirms that melatonin can suppress abnormal hyperexcitability in hippocampal CA1 neurons during the early stage of OGD, manifested as reduced evoked action potential firing frequency, improved action potential waveform, and prolonged evoked action potential latency. These findings provide partial kinetic characteristics of melatonin’s regulation of neuronal excitability.

## Data Availability

The original contributions presented in this study are included in the article. Further inquiries can be directed to the corresponding author.
